# Structural Modification Endows Small-Molecular SN38 Derivatives with Multifaceted Functions

**DOI:** 10.3390/molecules28134931

**Published:** 2023-06-22

**Authors:** Yi Dai, Meng Qian, Yan Li

**Affiliations:** 1 College of Pharmaceutical Science, Anhui Xinhua University, Hefei 230088, China; qianmeng@163.com (M.Q.); liyan@163.com (Y.L.); 2 Department of Chemistry, University of Science and Technology of China, Hefei 230026, China

**Keywords:** 7-ethyl-10-hydroxycamptothecin, structural modification, tumor targeting, theranostics

## Abstract

As a camptothecin derivative, 7-ethyl-10-hydroxycamptothecin (SN38) combats cancer by inhibiting topoisomerase I. SN38 is one of the most active compounds among camptothecin derivatives. In addition, SN38 is also a theranostic reagent due to its intrinsic fluorescence. However, the poor water solubility, high systemic toxicity and limited action against drug resistance and metastasis of tumor cells of SN38 indicates that there is great space for the structural modification of SN38. From the perspective of chemical modification, this paper summarizes the progress of SN38 in improving solubility, increasing activity, reducing toxicity and possessing multifunction and analyzes the strategies of structure modification to provide a reference for drug development based on SN38.

## 1. Introduction

**Figure 1:**
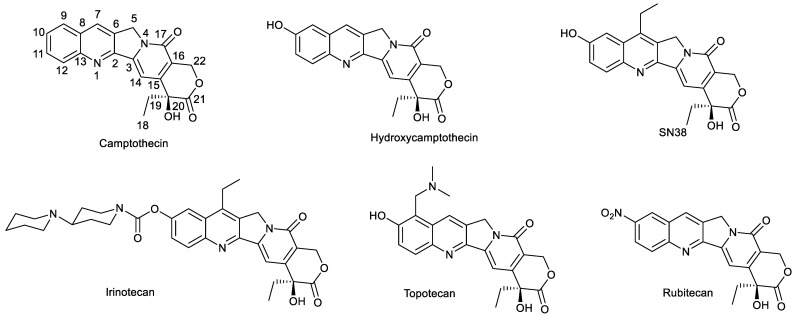
Camptothecin, hydroxycamptothecin, SN38 and clinical drugs based on camptothecin.

Natural products are an important source for developing chemotherapeutic agents. However, natural products, except for paclitaxel, are seldom used directly for the treatment of cancer in clinic due to their intrinsic defects such as poor water solubility, systemic toxicity, weak bioactivity, etc. Therefore, the structural modification of natural products is always a research focus for chemists and pharmaceutical scientists. For instance, many research projects are carried out to structurally modify vinblastine, tropolones, oridonin, etc. [1,2,3,4,5]. Camptothecin derivatives, another important source for the development of antitumor drugs, exert antitumor activity by inhibiting topoisomerase 1. Based on the skeleton of camptothecin, some drugs have been developed for the treatment of cancers, such as topotecan, irinotecan, rubitecan, etc. [6] (Figure 1). The research on camptothecin derivatives, especially 7-ethyl-10-hydroxycamptothecin (SN38), has always attracted much attention from medicinal chemists.

As an active metabolite of irinotecan, SN38 shares a similar anticancer mechanism (inhibition of topo 1) with other camptothecin derivatives and is considered to be one of the most potent antineoplastic agents among camptothecin derivatives. However, the deficiency of SN38, such as poor water solubility, instability, systemic toxicity and weak inhibitory effect on tumor resistance and metastasis, has greatly limited its clinical applications as an anticancer drug [7]. Therefore, structural modification of SN38 is in demand for new drug development. In recent years, a variety of structural modifications of SN38 for addressing the above problems have been reported. We searched the literature through SciFinder using 7-ethyl-10-hydroxycamptothecin as the substance identifier, and 3734 articles were obtained. Then, the articles were refined by document type (Journal and Letter) to obtain 2137 articles, followed by being refined again in accordance with publication year (2018–2023) to obtain 493 articles. We reviewed these articles and recapitulated them. Structural modification of SN38 was classified into five categories: (1) structural modification at position 10 of SN38; (2) structural modification at position 20 of SN38; (3) structural modification at position 10 and 20 of SN38; (4) structural modification at position 9 of SN38; and (5) lactone E ring-opening of SN38. These obtained SN38 derivatives exhibited multifaceted functions, as shown in Table 1. In addition, this also probes into the strategy of the current study concerned with the structural modification of SN38, providing a reference for the further development of SN38.

## 2. Modification of SN38 at C-10 Position

**Figure 2:**
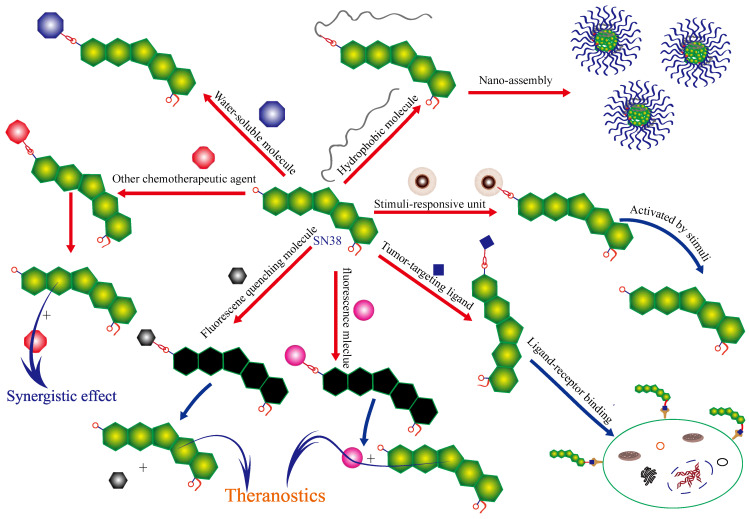
Modification of SN38 at C-10 position with small-molecule units to exert their functions.

The hydroxyl group at C-10 of camptothecin derivatives plays an important role in maintaining the stability of the parent structure and exerting anticancer activity [8,9]. Therefore, designing a prodrug of SN38 is one of the best design strategies due to the release of parent SN38 and other functional groups from the prodrugs. The small-molecule SN38 derivatives based on the modification at C-10 of SN38 account for a high proportion of all small-molecule SN38 derivatives. These small-molecule SN38 derivatives possess stimuli-responsive release of drugs, tumor targeting, synergism between different drugs, improvement of water solubility and theranostic function, as shown in Figure 2.

### 2.1. Stimuli-Responsive Release of Drugs

**Figure 3:**
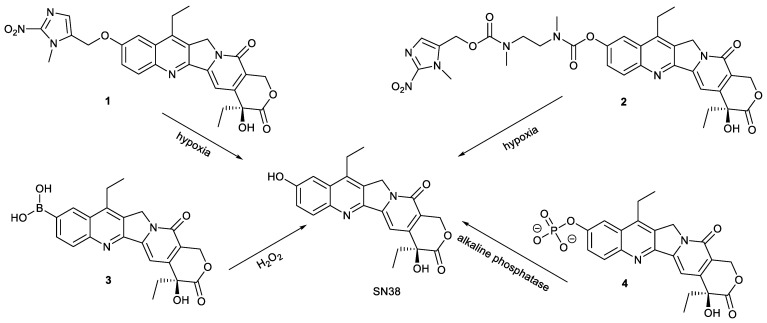
Endo-stimuli-responsive SN38 prodrugs based on modification at C-10 position of SN38.

Designing prodrugs capable of stimuli-responsive release is of great value for improving the selectivity of drugs to cancer cells to achieve high efficiency and low toxicity [10,11,12]. Modification of SN38 also follows the principle to utilize the special microenvironment of cancer cells for the design of SN38 derivatives. Upon cellular internalization, these SN38 derivatives can release the active parent SN38 in response to special stimuli in cancer cells. Compared with normal cells, the microenvironment of tumor cells is hypoxia, high hydrogen peroxide and high expression of esterase, etc. [13,14,15]. Based on these findings, compounds **1**–**4** were designed and synthesized (Figure 3). Compounds **1** and **2** both contain hypoxia-responsive moiety of (1-methyl-2-nitro-1H-imidazole-5-yl) methanol and can release the parent SN38 in responsive to hypoxia presented in cancer cells. The linkage between (1-methyl-2-nitro-1H-imidazole-5-yl) methanol and SN38 was ether bond for compound **1,** while bis-carbamate for compound **2**, which significantly affected their pharmacokinetic properties. Compound **1** was considered to be superior. The inhibitory effect of compound **1** on human colorectal cancer H460 cells and human colon cancer HT-29 cells was stronger than that of compound **2** due to its higher selectivity to hypoxia, even 10 folds stronger than that of evofosfamide [16].

Compound **3** was prepared by replacing the hydroxyl group at C-10 of SN38 with boric acid. Utilizing the high levels of H_2_O_2_ in cancer cells, compound **3** could be activated to release SN38. The inhibitory effect of compound **3** on topo 1 was stronger than that of SN38. The cell survival assay showed that compound **3** effectively inhibited the growth of human colon cancer HCT-15 cells, human breast cancer MCF-7 cells, human breast cancer MDA-MB-231 cells, human astroblastoma U87MG cells, human glioma U251 cells and HT-29 cells. The cytotoxicity of compound **3** against MCF-7 cells and U251 cells was comparable to that of SN38, but for HT-29 cells, the cytotoxicity of compound **3** was stronger than that of SN38. The in vivo experiment demonstrated compound **3** at a dose of 2.0 mg/kg significantly inhibited the tumor growth of U87MG tumor-bearing mice, indicating that the feasibility of boron acid being responsive to H_2_O_2_ [17].

Based on the high expression level of alkaline phosphatase on the membrane of human-induced pluripotent stem cells (hiPSCs) and neural stem cells [18], compound **4** containing phosphate at position 10 was designed. The high water solubility caused compound **4** not to be taken up effectively by normal neural cells and to show low cytotoxicity due to absence of alkaline phosphatase on the membrane of these normal cells. Compared with normal neural cells, hiPSCs and neural stem cells could make compound **4** be hydrolyzed to release SN38 of high penetration and cytotoxicity. Therefore, compound **4** can selectively eliminate proliferative hiPSCs and neural stem cells from neural cell mixtures. It is thus evident that simple phosphorylation of SN38 provided a cost-effective tool for decontaminating hiPSC-derived neurons [19].

**Figure 4:**
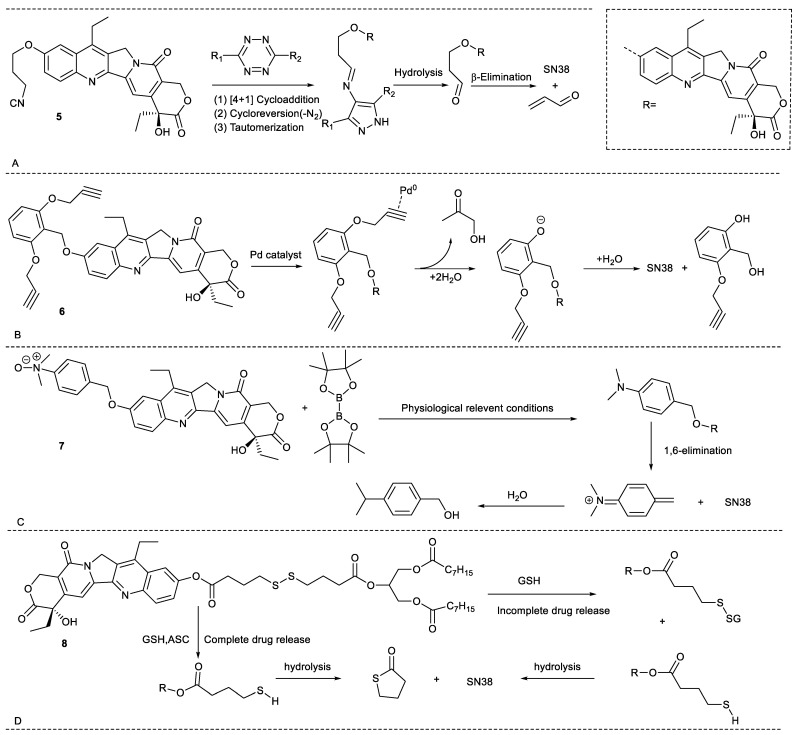
Release mechanism of compounds **5**–**8.** (**A**): Responsive to tetrazines, compound **5** underwent [4+1] cycloaddition, cycloreversion, tautomerization and β-elimination to release SN38; (**B**): Responsive to nanopalladium, compound **6** underwent palladium-mediated O-dealkylation to release SN38; (**C**): Responsive to bis(pinacolato)diboron, compound **7** underwent click reaction to release SN38; (**D**): Responsive to GSH and ASC, compound **8** underwent reduction and hydrolysis to release SN38.

Apart from endogenous substances in cancer cells, some exogenous substances can also activate SN38 prodrugs. For example, compound **5** was prepared through connecting the 3-isocyanopropyl group to C10-OH of SN38. The compound and tetrazines underwent dissociative bioorthogonal reactions to near-quantitatively release SN38, providing an alternative for the controlled release of SN38 (Figure 4A) [20]. Another example is compound **6**, obtained by the substitution of C10-OH of SN38 with 2,6-bis(propargyloxy)benzylgroup. Based on biorthogonal reactions, compound 6 cannot release SN38 in the absence of exogenous palladium, while it can release SN38 in the presence of exogenous palladium (Figure 4B). Hence, compared with the cytotoxicity of SN38, the cytotoxicity of compound **6** in the absent of exogenous palladium significantly decrease by 44-fold,16-fold and 35-fold for human colon cancer HCT-116 cells, human glioma U87 cells and U251 cells, respectively. Once co-existed with exogenous palladium, the compound showed cytotoxicity comparable to SN38. In addition, the combination of compound **6** and the Pd-activated 5-FU prodrug can synergistically inhibitthe growth of cancer cells through common palladium catalysis. These findings demonstrate that the combination of compound **6** with tumor targeting nanopalladium can realize the controlled release of SN38 in cancer cells and decrease toxic and side effects on other organs [21]. Another example was compound **7,** which was synthesized by introduction of benzylic alcohol containing a tertiary amino group in the form of N-oxide to SN38. The introduction of the group not only significantly enhanced the water solubility of SN38 (300-fold) but also weakened its cytotoxicity against human non-small-cell lung cancer A549 cells (10-fold). Upon exposure to bis(pinacolato)diboron, the compound was activated via a click reaction to release SN38 with fast reaction kinetics and complete cleavage both in vitro and in vivo (Figure 4C) [22]. These findings show that the bioorthogonal prodrug strategy presents significant advances.

Additionally, inspired by the specific absorption mechanism of triglyceride fat, compound **8** was synthesized by conjugation of SN38 with a triglyceride fat containing a disulfide bond. As a structured lip mimetic oral prodrug, compound **8** had a better oral bioavailability than that of SN38. When the compound was coadministrated with ascorbic acid (ASC), it could more efficiently release SN38 via activation by GSH and ASC, which provided a reference for the difficult-for-oral chemotherapeutics (Figure 4D) [23].

### 2.2. Tumor Targeting

**Figure 5:**
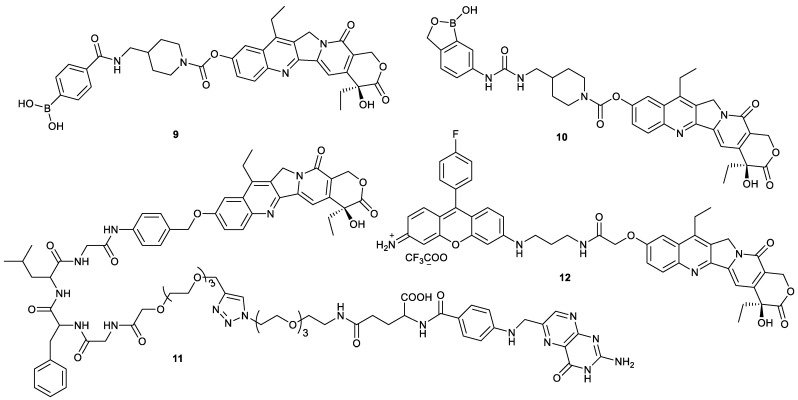
Structures of compounds **9**–**12**.

Based on the overexpressed receptors or polysaccharides on the surface of tumor cells, the relevant ligands are selected to decorate small-molecule drugs for achieving active targeting conjugates [24,25]. This strategy has also been successfully applied in the design of the tumor-targeting SN38 derivatives. Sialic acid is expressed highly on the surface of cancer cells and has a high affinity with phenylboronic acid [26,27]. Therefore, introduction of phenylboronic acid moiety into SN38 can prepare the tumor-targeting SN38 derivatives, such as compounds **9** and **10** (Figure 5). Compared with uptake of irinotecan, the uptake of compounds **9** and **10** by human hepatoma HepG2 cells was enhanced 3-fold and 7-fold, respectively. However, the cytotoxicity of compounds **9** and **10** was comparable to that of irinotecan due to the urethane linker between phenylboronic acid and SN38 [28]. The linker was too stable to effectively release SN38, indicating that more attention should be paid to the linker between ligands and drugs for the highly efficient release of parent drugs.

Compound **11** was a dual-targeting prodrug (Figure 5). The tumor-targeting ability and parent drug release were both taken into consideration for the design of compound **11**, which contained a folate as a tumor-targeting moiety, due to the overexpression of folate receptors on the tumor surface, and a tetrapeptide Gly-Phe-Leu-Gly (GFLG) as an enzyme-responsive substrate, which was responsive to cathepsin B, which is highly expressed in tumor cells and beneficial for enhancement of water solubility. For human hepatoma SK-Hep-1 cells, human cervical cancer HeLa cells and human cervical cancer SiHa cells with high folate receptor expression, the IC_50_ value of compound **11** was 2–3 µM, while it has little effect on A549 cells and human bronchial epithelioid 16HBE cells with a negative folate receptor, indicating its good cell selectivity [29]. Compound **12** was prepared by decoration of SN38 with Kyoto probe 1 that selectively labels human-induced pluripotent stem cells (hiPSCs) (Figure 5). Compound **12** can selectively induce the death of hiPSCs, which provides a good strategy for eliminating the residual undifferentiated stem cells to ensure safety and decrease the tumorigenic risk in stem cell therapy [30].

### 2.3. Improvement of Water Solubility

**Figure 6:**
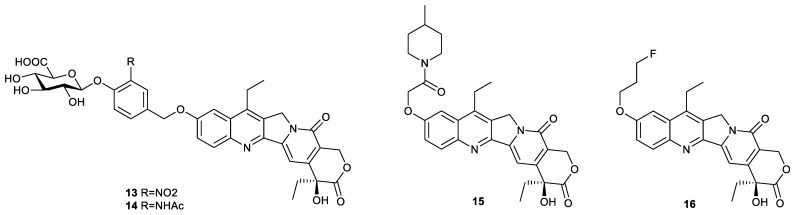
Structures of compounds **13**–**16**.

To address the issue of poor water solubility of SN38, there are mainly two methods reported in the literature: one is insertion of water-soluble moiety into SN38, and the other is the preparation of more hydrophobic SN38 derivatives for facilitating nanodelivery via encapsulation (Figure 5). For example, compound **13** and compound **14** were both combinations of SN38 with glucuronic acid via a self-immolative linker, and they were responsive to β-glucuronidase to release SN38 (Figure 6). The IC_50_ values of compound **13** and compound **14** against HeLa cells were 0.6 µM and 2 µM, respectively, in the absence of β-glucuronidase, while 59 nM and 82 nM, respectively, in the presence of β-glucuronidase, demonstrating the self-immolative capacity of the linker [31]. Compound **15** and compound **16** also had higher water solubility than SN38 (Figure 6). The former was the insertion of 4-methylpiperidine into SN38, and the latter was the introduction of fluoroalkyl into SN38. The solubility of compound **15** reached 5.73 µg/mL in water. Moreover, compound **15** had stronger cytotoxicity toward A549 cells, HCT-116 cells, human colon carcinoma LoVo cells, human colon carcinoma Colo205 cells, HT-29 cells and HepG2 cells than SN38. The in vivo experiment showed that compound **15** significantly inhibited tumor growth in A549 tumor-bearing mice at dosages of 0.4 and 2.0 mg/kg and exhibited minimum lethal doses comparable to those of irinotecan [32]. The water solubility of compound **16** was enhanced by 17-fold in phosphate-buffered saline compared with SN38. Though the in vitro cytotoxicity of compound **16** toward prostate cancer, PC-3 cells were around half that of SN38, and compound **16** had a higher inhibitory effect on tumor growth in PC-3 tumor-bearing mice than SN38, even at lower dosage [33]. More importantly, fluoride can be tracked in cells by NMR to investigate its disposition, which deserves further study.

**Figure 7:**
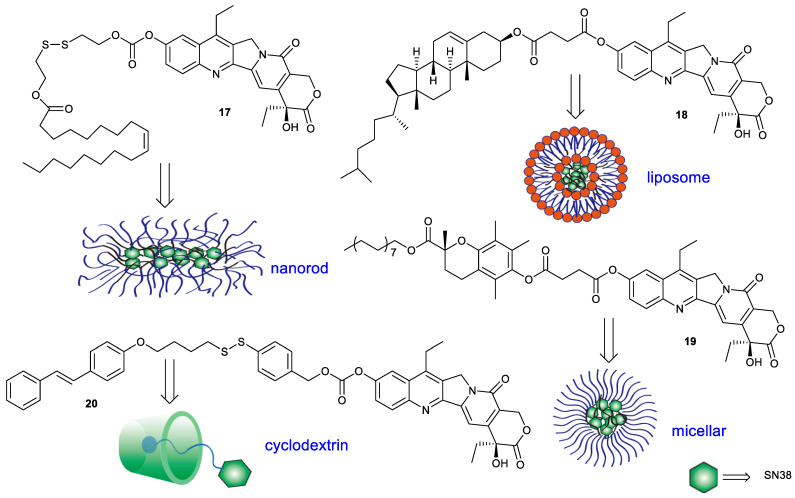
Modification of SN38 at position 10 with hydrophobic groups and their transportation.

More hydrophobic SN38 derivatives can be encapsulated easily by carrier material to facilitate efficient SN38 delivery (Figure 7). Compound **17** was synthesized by conjugating SN38 with oleic acid via disulfanyl–ethyl carbonate. The encapsulation of compound **17** with DSPE-mPEG2000 could form nanorods and demonstrate colloidal stability. Upon exposure to thiols, the nanorods can rapidly release SN38. For instance, the nanorods could release 1% SN38 within 1 h in phosphate buffer (pH 7.4), versus 100% in 10 mM DTT. The in vitro cytotoxicity of the nanorods toward mouse breast carcinoma 4T1 cells, mouse melanoma B16-F10 cells and mouse colon cancer CT26 cells was comparable to free SN38 and around 90-fold more potent than irinotecan. In addition, the nanorods had a higher inhibitory effect on tumor growth in CT26 tumor-bearing mice than irinotecan [34]. Compound **18** was an esterase-responsive prodrug which was prepared by conjugation of SN38 with cholesterol via succinic acid. The introduction of cholesterol endowed compound **18** with elevated miscibility with liposomal compositions and was beneficial for the formation of nanoliposomes. The resulting nanoliposomes with the drug loading of 9.79% exhibited a size of around 78 nm and a zeta potential of around −28.9 mV. The in vitro cytotoxicity of the nanoliposomes against A549 cells, human lung cancer PC-9 cells, LoVo cells and MCF-7 cells was slightly weaker than that of SN38 alone, while stronger than that of irinotecan. The tumor inhibitory rate of the nanoliposomes in A549 tumor-bearing mice was enhanced by around 1.5-fold compared with that of irinotecan. Moreover, the form of drug delivery significantly improved drug tolerability [35]. Compound **19**, a hybrid of SN38 and vitamin E analogue, was synthesized using succinic acid as a linker. Due to high lipid solubility, compound **19** could form nanomicelles with Tween 80 and PEG200. The resulting nanomicelles had much stronger cytotoxicity against human ovarian cancer A2780 cells, A549 cells, HT-29 cells and HepG2 cells than irinotecan [36]. Compound **20** was prepared by conjugation of SN38 with azobenzene moiety via a redox-sensitive self-immolative linker containing a disulfide bond. The hydrophobic compound was delivered through inclusion of beta-cyclodextrin, which widely exhibited applications in many areas, such as the pharmaceutical industry, food technology, cosmetics and dyes, etc., due to its nontoxicity, aqueous solubility, biodegradability and biocompatibility [37]. Compound **20** could be wrapped by beta-cyclodextrin to further self-assemble into tubular or vesicular. The obtained tubular or vesicular could release the parent drug in two ways: one was dependent on the change in cis–trans isomerization of azobenzene exposed to ultraviolet light, and the other was dependent on the reduction in disulfide bond by GSH [38]. The design ideas for these compounds provide a reference for the delivery of other hydrophobic drugs.

### 2.4. Synergism between Different Drugs

**Figure 8:**
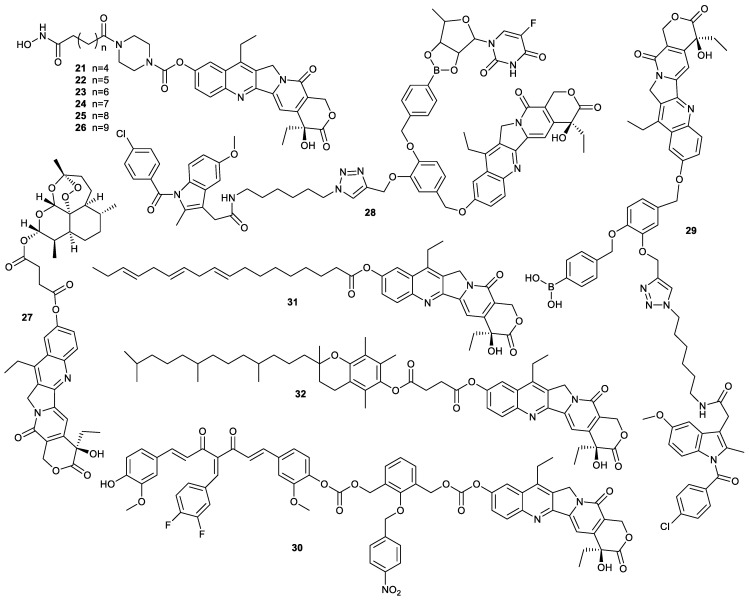
Structures of compounds **21**–**32**.

A single drug is generally ineffective for the treatment of some cancers due to the complexity of the initiation and progression of human tumors. Designing multitarget drugs for the treatment of cancer is an important strategy for developing novel antitumor drugs [39]. The introduction of moieties capable of combating cancers in SN38 to prepare a prodrug of SN38 is beneficial for the synergism of different drugs (Figure 8). Upon cellular internalization, this type of prodrug can release parent drugs to synergistically inhibit the growth of cancer cells. Aberrant histone deacetylases (HDACs) have a significant correlation with tumors. Hydroxamic acid derivatives can suppress the growth of cancer cells via inhibition of histone deacetylases. Based on these findings, compounds **21**–**26** were prepared by the introduction of hydroxamic acid moieties into SN38. These compounds were stable in buffers or plasma. However, their inhibitory effect on HDAC was weaker than that of SAHA and was concerned with the length of the carbon chain in hydroxamic acid moieties; the stronger the inhibitory effect on HDAC6, the longer the carbon chain. Due to the difficulty in the release of parent SN38, these compounds exhibited lower cytotoxicity toward A549 cells and HCT-116 cells than SN38 and SAHA, indicating that ensuring the release of parent SN38 was crucial in the design of SN38-based prodrugs [40].

Compound **27**, a hybrid of SN38 and artesunic acid, significantly suppressed the growth of the yeast *Saccharomyces cerevisiae* EKY3 strain via inhibition of the overexpressed human topo 1. The IC_50_ values of compound **27** against human melanoma RPMI7851 cells and human melanoma SK-MEL 24 cells were 0.05 ± 0.02 µM and 0.32 ± 0.03 µM, respectively, while those of SN38 were 0.51 ± 0.26 µM and 0.48 ± 0.07 µM, demonstrating that compound **27** had more cytotoxicity than SN38 [41]. Compound **28** was another prodrug formed by conjugation of SN38 with 5′-deoxy-5-fluorouridine (5′-DFUR) and indomethacin (IMC). Upon cellular internalization, compound **28** containing borate ester was responsive to H_2_O_2_ to release SN38, 5′-DFUR and IMC to inhibit topo 1, thymidylate synthase and COX2, respectively. Due to a strong synergetic effect, compound **28** showed higher cytotoxicity toward COX2-positive human pancreatic carcinoma MIA Paca-2 cells than compound **29,** only containing the IMC unit and SN38 unit, indicating that the hybrid containing multiple drugs deserved further investigation. In addition, compound **28** had marginal inhibitory effect on COX2-negative human intestinal Caco-2 cells, exhibiting the tumor-targeting ability [42].

Compound **30** was another hybrid of SN38 and 3,4-difluorobenzylidene curcumin, which could be activated in hypoxic conditions to release SN38 as an inhibitor of topo 1 and 3,4-difluorobenzylidene curcumin as an inhibitor of cancer stem-like cells (CSCs). The in vitro and in vivo experiments showed that the compound could inhibit the metastasis and multidrug resistance of triple-negative breast cancer via eradication of CSCs and downregulation of multidrug resistance [43].

Apart from SN38-based hybrids, codelivery of SN38 derivatives with other chemotherapeutic agents also can create a synergistic effect. For example, SN38 was esterified with α-linolenic acid to obtain compound **31**, then compound **31** and the combretastatin-A4 derivative were encapsulated by mPEG_5k_−PLA_16k_ to prepare nanoparticles. The resulting nanoparticles could inhibit the growth of cancer in a cocktail manner and showed much stronger inhibitory effect on HT-29 cells and HT-29 tumor-bearing mice than free SN38 or its encapsulation. In addition, the nanoparticles could inhibit the metastasis of HUVEC [44]. Another example was compound **32**, formed by the conjugation of SN38 with α- tocopherol. The as-prepared prodrug could block detrimental intercellular connections, which were the driving force for the acquired drug resistance and proliferative burst. Compared with MCF-7 cells, the paclitaxel-resistant MCF-7 cells (MCF-7/PTX) absorbed compound **32** more efficiently, which led to the rapid release of parent SN38 to exerted higher cytotoxicity. Similar results were exhibited in the cisplatin-resistant HeLa cells and HeLa cells. However, due to strong hydrophobicity, free compound **32** had difficulties circulating in blood, which could be overcome by encapsulation of compound **32** with amphiphilic block copolymers PEG_5k_-b-PMASSV. Besides high drug-loading capacity and stability, the obtained nanoparticles had significant anticancer efficacy against both drug-resistant cancer cells and drug-sensitive cancer cells via the above-mentioned antitumor mechanism [45]. Hence, exploiting nanodelivery systems is an alternative for hydrophobic agents.

### 2.5. Theranostics

**Figure 9:**
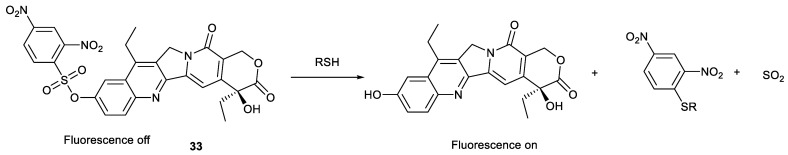
Fluorescence imaging mechanism of compound **33**.

Owing to the demand for precision medicine, theranostics is a new trend of developing anticancer drugs, especially for small-molecular theranostics which can be prepared easily and have high tumor-targeting ability and low side effects [46]. Since the introduction of a strong electron-withdrawing group into SN38 at C-10 position will quench or attenuate the intrinsic fluorescence of the conjugated fluorophores via donor-excited photoinduced electron transfer (d-PeT), it is convenient for developing the “turn-on” theranostics based on SN38. As an electron-withdrawing group, the sulfonic group was usually used as the attachment group, such as the sulfonic esters of N-oxyimides, known for a wide range of biological activities [47]. Compound **33**, a conjugate prepared by the introduction of 2,4-dinitrobenzene sulfonyl into SN38 at C-10 position, could not produce the fluorescence due to d-PeT. Upon exposure to GSH or some sulfhydryl compounds, compound **33** could release SN38 and produce its intrinsic fluorescence to simultaneously exert therapy and diagnosis via the hydrolyzation of sulfonate moiety (Figure 9). The in vitro cytotoxicity showed that the inhibitory effect of compound **33** on HeLa cells and m-Cherry + OCSC1-F2 cells were comparable to those of SN38 [48].

**Figure 10:**
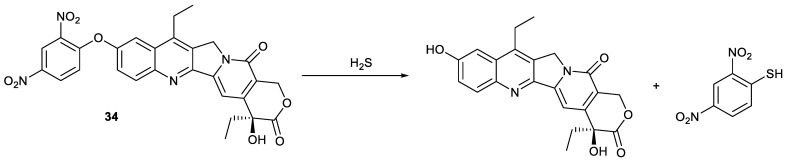
Fluorescence imaging mechanism of compound **34**.

Compound **34** was synthesized by the introduction of dinitrobenzene into SN38, and its intrinsic fluorescence and activity was significantly quenched by the dinitrobenzene group. Like compound **33**, the compound could be responsive to hydrogen sulfide to release SN38 to obtain significantly enhanced fluorescence and activity for real-time monitoring SN38 release and antiproliferation, respectively, which was exhibited in HCT-116 cells and 4T1 cells (Figure 10). It showed stronger cytotoxicity against cancer cells than irinotecan, while exhibiting marginal cytotoxicity against normal human embryonic kidney HEK-293T cells, which was attributed to overexpression of H_2_S in cancer cells in comparison with normal cells [49].

**Figure 11:**
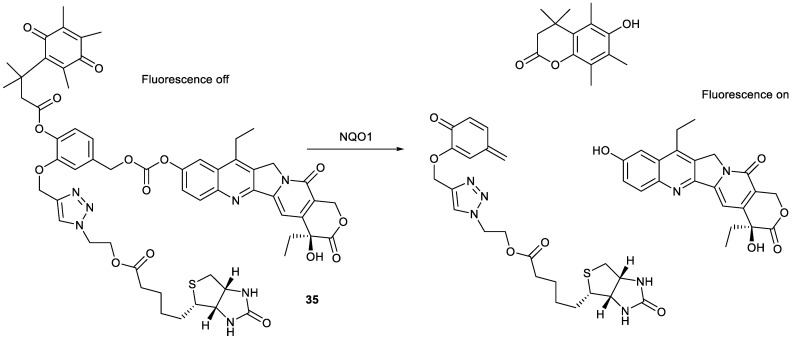
Fluorescence imaging mechanism of compound **35**.

Theranostics with tumor-targeting ability are desirable for the precise diagnosis and treatment of cancer. Based on the overexpression of NAD(P)H:quinone oxidoreductase-1 (NQO1) and the biotin receptor in cancer cells, compound **35** was designed by the introduction of both biotin and quinone propionic acid into SN38 at C-10 position. Due to the insertion of quinone propionic acid, the resulting compound could not produce fluorescence in comparison with SN38. Upon exposure to NQO1, compound **35** could be trigged via quinone propionic acid unit to release parent SN38 as a theranostic agent (Figure 11). The biotin moiety endowed compound **35** with the tumor-targeting ability. For example, compound **35** had significant cytotoxicity against A549 cells and HeLa cells, comparable to SN38, while it demonstrated a marginal effect on human normal fibroblast WI-38 cells and human normal fibroblast BJ cells. Encouragingly, the tumor-targeting ability of compound **35** was verified in the A549 tumor-bearing mice model, showing that compound **35** was mainly accumulated in the tumor organ and had stronger antitumor activity than SN38 [50].

**Figure 12:**
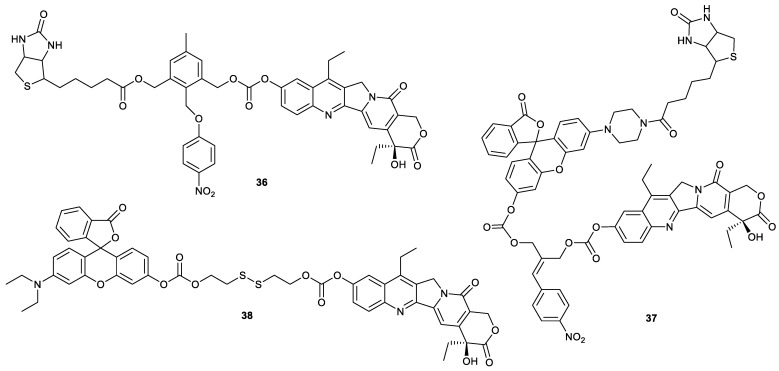
Structures of compounds **36**–**38**.

Another theranostic agent was compound **36** (Figure 12)**,** containing biotin as the tumor-targeting unit and 4-nitrobenzene as the hypoxic-responsive unit. Compound **36** was activated in the presence of nitroreductase and NADH in the hypoxic environments of solid tumors, where it was reduced to release SN38 as a theranostic agent. Compound **36** showed a much stronger cytotoxicity against A549 cells and HeLa cells than against normal BJ cells and WI-38 cells, similar to compound **35**. In addition, compound **36** had stronger antitumor activity than SN38 in a HeLa tumor-bearing mice model. The fluorescence imaging demonstrated compound **36** could penetrate tissue deeply and be specially accumulated in tumor tissue and activated by hypoxia to release SN38 [51]. Compound **37** was prepared by the introduction of rhodol into compound **36** (Figure 12). There was only a difference in the fluorescence imaging unit between compound **36** and compound **37**. The former was a utilization of SN38 as a fluorophore, and the latter was a utilization of rhodol as a fluorophore. Responsive to hypoxia, compound **37** released SN38 to inhibit topo 1 and rhodol to track the distribution of drug. The biotin moiety endowed compound **37** with enhanced cytotoxicity against HeLa cells and decreased cytotoxicity against mouse embryonic fibroblast NIH3T3 cells [52].

Apart from the insertion of a tumor-targeting unit into SN38 derivatives, encapsulation of SN38-based theranostic agents with carrier material capable of targeting tumors can also render the tumor-targeting ability of theranostics possible. For example, though compound **38** formed by conjugation of SN38 with rhodol via disulfide bond did not have tumor-targeting ability (Figure 12), the “turn-on” theranostic prodrug was encapsulated by biotinylated poly(vinyl alcohol) to form a tumor-targeting nanoparticle. Upon cellular internalization, compound **38** was activated by GSH to release SN38 and rhodol to induce apoptosis and monitor the delivery of drugs, respectively. These nanoparticles showed remarkably higher stability in blood serum and much stronger selectivity to cancer cells. The nanoparticles significantly inhibited the proliferation of HeLa cells in a dose-dependent manner while showing only marginal cytotoxicity toward NIH3T3 cells [53].

### 2.6. Other Functions

For exploiting SN38 derivatives with higher anticancer activity, compound **39** and compound **40** were synthesized. They showed a strong inhibitory effect on tumor cells, especially compound **40**, with IC_50_ values of 0.002, 0.003 and 0.081 µM toward human B lymphoma Raji cells, HCT-116 cells, A549 cells and LoVo cells, respectively. Apart from stronger antitumor activity than SN38, compound **40** could be administrated orally with the bioavailability of 22.4% for mice and significantly suppress the HCT-116-xenograft tumor growth in the nude-mice model at dosages of 0.5, 2.0 and 8.0 mg/kg. When compound **40** was administered intraperitoneally to Raji tumor-bearing mice at dosages of 2.0 and 4.0 mg/kg, Raji xenograft tumor growth was completely inhibited. In addition, the minimum lethal doses of compound **40** was two-fold that of SN38, indicating that compound **40** had much higher safety than SN38 [54]. If the substitution of C10-OH of SN38 with fluorine was carried out, compound **41** was obtained. As a topo 1 inhibitor, compound **41** showed more potent in vitro cytotoxicity against serval types of cancer cells (human colon cancer SW480, human pancreatic cancer SW1990, human hepatoma Hep3B, HepG2, A549, A2780, HeLa and human cholangiocarcinoma QBC cells) and inhibitory effect on tumor growth in HCC and ICC mouse models than topotecan, while showing only marginal toxicity to mouse noncancerous tissues [55].

Compounds **42**–**44**, whose IC_50_ values for human leukemia K562 cells were higher than that of SN38, were prepared by the introduction of 2-nitrobenzene, 3-nitobenzene or 4-nitrobenzene into SN38 via ether bond [56]. Compounds **45**–**51** reported by SQ Zhang and coworkers had an inhibitory effect on A549 cells and HCT-116 cells comparable to that of SN38 [32]. Compounds **52**–**57** showed slightly lower cytotoxicity toward PC-3 cells than SN38 [33]. Compound **58** was synthesized by the introduction of amino acid to SN38. The compound showed higher cytotoxicity against human colon cancer SW1116 and human colon cancer LS174T cell than irinotecan [57].

The rapid release of the parent SN38 from SN38-based prodrug is the key criterion for designing these drugs, which deserves more attention. For example, compound **59**, formed by conjugation of SN38 with 2-(aminomethyl)pyrrolidine via carbamate, was able to rapidly release SN38 in a self-immolative manner. When compound **59** was incubated in a DMSO/acetate buffer mixture (pH 5.5) at 37 °C, it released SN38 with a half-life (t_1/2_) of 1.6 h, more rapidly than compound **60** (t_1/2_ = 10.6 h) and compound **61** (t_1/2_ = 38.9 h) [58].

**Figure 13:**
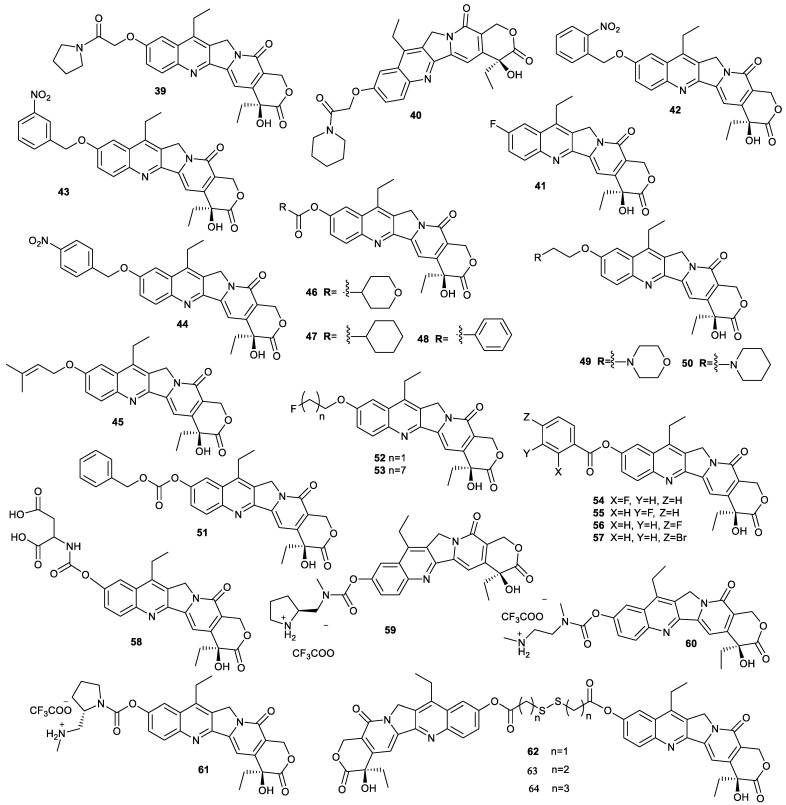
Structures of compounds **39**–**64**.

Compounds **62**–**64** were SN38 homodimeric prodrugs with different linkers containing disulfide bonds (Figure 13). These compounds exhibited different chemical and self-assembly stability due to different linker length. Among them, compound **64** possessed good chemical and self-assembly stability. The homodimeric prodrug nanoassemblies (HDPNs) formed by compound **64** had a higher inhibitor effect on 4T1 cells than SN38. Additionally, compound **64** significantly accumulated in tumor tissue to exert its antitumor activity in 4T1 tumor-bearing mice [59]. These findings provided a reference for the design of HDPNs.

## 3. Modification of SN38 at C-20 Position

Ensuring the structural integrity of camptothecin analogues is very important for the maintenance of antitumor activity. The hydrolysis of the E-ring of camptothecin derivatives will give rise to decreased antitumor activity [60]. It is well established that a prodrug with ester bond as a linker can be hydrolyzed to release a parent drug by esterase in cells. The esterification of C_20_-OH of camptothecin derivatives not only enhances their stability due to high steric hindrance but also endows them with multifunction, attributed to the introduction of other molecules [61]. The structural modification of SN38 at C-20 position has made progress and obtained some derivatives with the enhancement of synergism, tumor-targeting activity or solubility in recent years.

### 3.1. Synergism between Different Drugs

Owing to the complexity of the initiation and progression of human tumors, the combination of different drugs for the treatment of cancer is an effective strategy. Histone deacetylase inhibitors can sensitize chemotherapeutic drugs, including camptothecin-based drugs [62,63]. For example, compounds **65**–**68** synthesized by conjugation of SN38 with SAHA via the amino acids of different chain length demonstrated that their cytotoxicity against A549 cells and HCT-116 cells increased with decreasing amino acid chain length, which was consistent with the results of their release rate of parent drugs from these prodrugs, that is, the release rate increased gradually with decreasing amino acid chain length. While compound **65**, the strongest antitumor compound among these prodrugs, showed a slightly weaker antitumor activity than SN38, it exhibited a significantly stronger cytotoxicity than SAHA [64]. The hydroxamic acid moiety of SAHA played a key role in inhibiting histone deacetylase. The direct conjugation of hydroxamic acid moiety with SN38 was carried out to finish the synthesis of compounds **69**–**75**. The inhibitory effect of these compounds on HDAC decreased with the increased chain length of C-20 position and was slightly lower than that of SAHA. Though these compounds demonstrated lower inhibition of HDAC than SAHA, they all showed higher cytotoxicity than SAHA, especially compound **75**, which could rapidly release SN38. These findings indicated that these as-prepared compounds exhibited synergistic antitumor activity by inhibiting both topo 1 and HDAC [40].

### 3.2. Tumor Targeting

The enhancement of tumor-targeting ability is beneficial for increasing antitumor activity and decreasing side effects. The decoration of SN38 with tumor-targeting units such as biotin, folic acid, small peptide and antibody is an effective strategy to enhance the tumor-targeting ability of chemotherapeutic agents [24,25]. For example, trastuzumab deruxtecan, an antibody–drug conjugate based on camptothecin, was approved by the US Food and Drug Administration for the treatment of several cancers [65]. SN38 was conjugated with iRGD (small cyclic peptidic motif, CRGDK/RGPD/EC) via disulfide bond to prepare compound **76**. The introduction of iRGD not only enhanced the water solubility of compound **76** but also inhibited cancer cell metastasis by targeting α_v_ integrins and neuropilin-1 overexpressed on the surface of several kinds of cancer cells [66,67]. Upon cellular internalization, compound **76** was responsive to GSH to rapidly release SN38 and iRGD. The inhibitory effect of compound **76** on high metastatic human hepatocellular carcinoma HCC-LM3 cells, human hepatocellular carcinoma BEL-7402 cells, HepG2 cells and A549 cells was 1–2 orders of magnitude higher than that of irinotecan, and its antimetastasis was also demonstrated in HCC-LM3 cells. Additionally, the iRGD released from compound 67 could block the metastasis of HCC-LM3 cells. The antiproliferation and antimetastasis of compound **76** was also verified to be stronger than that of irinotecan in HCC-LM3 tumor-bearing mice [68].

The introduction of heat shock protein 90 (HSP90) inhibitors NVP-AUY922 and SNX-5422 into SN38 prepared compounds **77**–**80**, which were targeted to cancer cells via extracellular HSP90-mediated endocytosis. These compounds showed excellent stability in buffers at pH 6.0 and 7.4 and in human plasma. However, the in vitro HSP90 binding affinity assay showed that conjugation of SNX-5422 with SN38 had negligible impacts on HSP90 activity, while conjugation of NVP-AUY922 with SN38 did not affect the binding affinity of the hybrids to HSP90, indicating that compounds **79** and **80** deserved further investigation, especially compound **79**, which showed strong cytotoxicity against A549 cells, HCT-116 cells, MIA-Paca-2 cells and human pancreatic cancer Capan-1 cells with IC_50_ values of 56 nM, 22 nM, 46 nM and 36 nM, respectively. In addition, compound **79** also showed strong antitumor activity in HCT-116 tumor-bearing mice and Capan-1 tumor-bearing mice, indicating that compound **79** is a promising new candidate for cancer therapy [69].

In regard to the overexpression of glucose transporters in cancer cells, compounds **81**–**83** were synthesized by conjugation of SN38 with glucose via click reaction. The introduction of glucose to SN38 simultaneously enhanced water solubility and tumor-targeting ability of the SN38 derivatives. Among them, compound **82** exhibited high cytotoxicity and selectivity for HCT-116 cells, which was also demonstrated in an HCT-116 xenograft model [70]. The design idea is also applicable to structural modification of other hydrophobic chemotherapeutic agents.

### 3.3. Improvement of Solubility

The introduction of water-soluble groups into SN38 can enhance the water solubility of SN38. For example, compound **84**, synthesized by the introduction of sulfonyl amidine into SN38, had higher water solubility than SN38. The cytotoxicity of compound **84** against A549 cells, oral epithelial carcinoma KB cells and multidrug-resistant KB cells was significantly stronger than that of irinotecan. In addition, compound **84** could reverse the multidrug resistance of KB cells [71]. Compound **85** was obtained by the decoration of SN38 with L-α-glycerophosphorylcholine. This compound could self-assembled into spherical liposomes with a size of ~140 nm, a potential of −21.6 ± 3.5 mV and a loading rate of 65.2%. The resulting liposomes were stable in a neutral environment but degraded to release SN38 in a weakly acidic condition. Moreover, the liposome had a longer blood retention time. After endocytosis, compound **85** showed strong cytotoxicity, comparable to that of SN38 [72].

**Figure 14:**
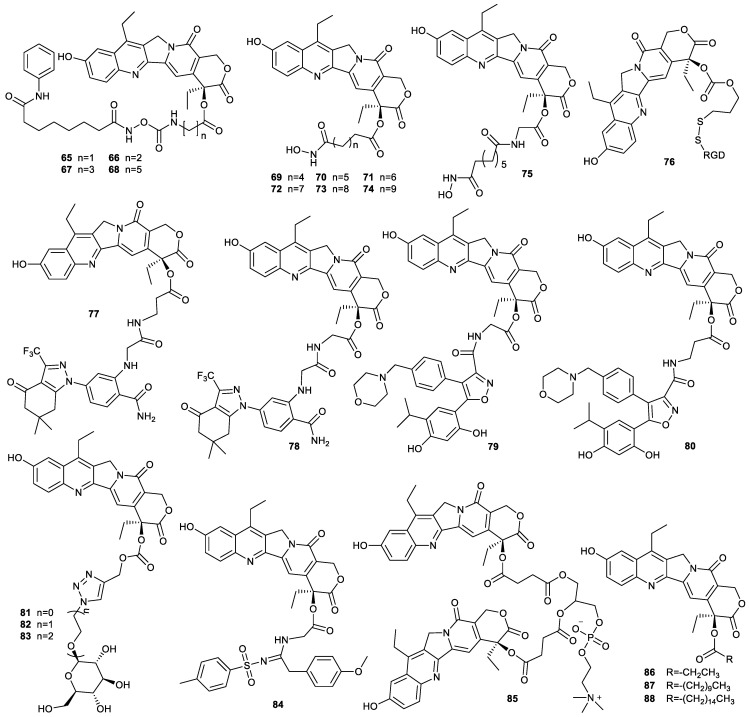
Structures of compounds **65**–**88**.

Another strategy for improving the solubility of SN38 is the insertion of long-chain fatty acids into SN38, which can improve solubility of the as-prepared compound in lipid excipients. For example, the solubility of compounds **86**–**88** in long-chain triglycerides was increased up to 444-fold (Figure 14). Their stability was controlled, and transmucosal permeability was enhanced. These compounds were stable in simulated gastric fluids but exhibited different rates of hydrolysis in simulated intestinal fluids (in the presence of enzymes) depending on the alkyl chain length [73]. Due to improvement in transepithelial drug transport and cellular uptake, these compounds were beneficial for oral administration.

## 4. Modification of SN38 at Both C-10 Position and C-20 Position

### 4.1. Tumor Targeting

Two methods are reported to prepare tumor-targeting SN38 derivatives decorated at both C-10 position and C-20 position. The front method is that C-10 is confined via insertion of a small group, and C-20 is conjugated with a tumor-targeting moiety, and the behind method is the reverse. Compound **89** was synthesized by the introduction of methoxymethyl ether at C-10 position and an arginine–glycine–aspartic acid–lysine (RGDK) peptide at the C-20 position of SN38. It could be targeted to the neuropilin-1 receptor and responsive to GSH. In addition, compound **89** could self-assemble into stable micelles with a hydrodynamic diameter around 110 nm and a fixed drug loading rate of 35%. The micelles prolonged the retention time in plasma and enhanced accumulation in the tumor tissue of compound **89**. Though the in vitro cytotoxicity of compound **89** against A549 was comparable to that of SN38, it showed much higher antitumor activity in a A549 xenograft mice model than irinotecan [74]. Compounds **90** and **91** containing phosphoramidites could be conjugated with aptamer automatically by solid-phase synthesis to prepare aptamer–SN38 conjugates with the tumor-targeting ability. For example, the conjugation of the two compounds with aptamer sgc8 could target the as-prepared compounds to HCT-116 cells. Moreover, as prodrugs, they also could release parent SN38 to induce apoptosis of cancer cells [75].

### 4.2. Theranostics

Exploiting SN38 derivatives with theranostic function is based on the intrinsic fluorescence of SN38. The direct conjugation of SN38 with 2,4-dinitro-benzene sulfonyl (DNS) at C-10 position causes the original fluorescence quenching, such as compounds **92** and **93**. In addition, compounds **92** and **93** had a quaternary ammonium moiety and sodium sulfonate moiety at C-20 position, respectively. Compound **92** could be fully threaded into the hydrophobic cavity of pillar [5]arene to form a supramolecular amphiphilic structure which self-assembles into nanoparticles, with an average hydrodynamic diameter of 253 nm and an encapsulation efficiency of 92.2%. Upon exposure to the high level of GSH in cancer cells, the nanoparticles could release SN38 to exert its antitumor activity and track its distribution in tissues due to the cleavage of their disulfide bonds and the DNS groups. Though compound **92** exhibited cytotoxicity against MCF-7 cells and HepG2 cells comparable to SN38, it demonstrated much lower cytotoxicity against NIH3T3 cells than SN38, indicating that compound **92** had excellent selectivity to cancer cells [76]. Compound **93** was encapsulated by another pillar [5]arene with tetraphenylethene functionalized on the bridged methylene group of the pillararene skeleton. Due the aggregation-induced emission (AIE) of pillar [5]arene and the intrinsic fluorescence of SN38, upon cellular internalization, the inclusions were responsive to GSH in cancer cells to release SN38 to inhibit cell proliferation, accompanied by fluorescence change from dim yellow to brilliant yellow for tracking their tumor tissue distribution. The inclusions showed their theranostic function in MDA-MB-231 cells and MDA-MB-231 tumor-bearing mice. The cytotoxicity of the inclusions toward MDA-MB-231 cells were stronger than that of SN38. Moreover, the inclusions showed marginal cytotoxicity against normal human embryonic lung fibroblast MRC-5 cells, as the pillar [5]arene [77].

### 4.3. Other Functions

The designing of compound **94** mainly paid more attention to the effective release of SN38. Compound **94**, incubated with a split BS2 esterase from *Bacillus subtilis*, could be effectively inverted into SN38 with a conversion rate of >95% and showed potent cytotoxicity against MDA-MB-231 cells in the presence of BS2, albeit with structural modification at both the C-10 position and C-20 position of SN38 [78]. The aim of designing compound **95** was to enhance the water solubility of SN38. Hence, phosphate and sulfonylamidine moieties were selected to conjugate with SN38 at C-10 position and C-20 position for preparation of compound **95**. Compound **95** exhibited 30–100-fold higher cytotoxicity against KB cells and multidrug resistant KB cells than irinotecan, demonstrating that compound **95** could reverse the multidrug resistance of KB cells [71]. Compounds **96**–**113** was synthesized by substitution of C10-OH with fluorine and esterification of C20-OH of SN38. Among them, compound **106** showed broad-spectrum cytotoxicity toward HepG2 cells, SW480 cells, A2780 cells and Hucct1 cells with IC_50_ values of 0.03, 0.09, 0.22 and 0.32 µM, respectively. In addition, compound **106** showed higher selectivity to cancer cells than topotecan. For example, the ratio of IC_50_ values to normal human hepatic LO2 cells and HepG2 cells was 113.20 for compound **106**, while 85.60 for topotecan. The high antitumor activity and low side effects of compound **106** were verified in a HepG2 tumor-bearing mouse model [79]. Due to the presence of fluorine, compound **106** in cells can be tracked by NMR, which merits further investigation.

**Figure 15:**
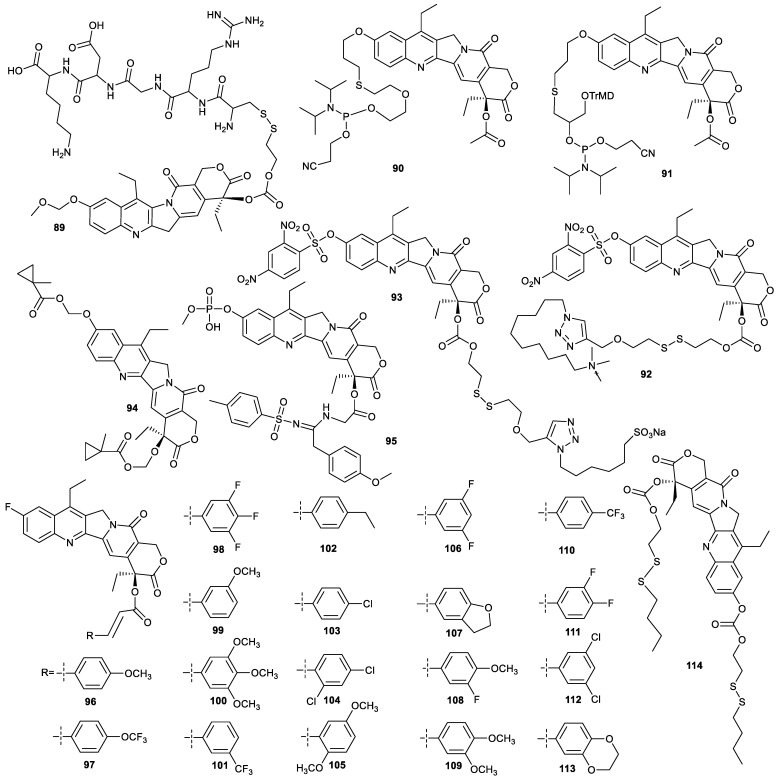
Structures of compounds **89**–**114**.

The esterification of SN38 at position 10 and position 20 with fatty acid containing a disulfide bond was used to prepare more hydrophobic compound **114** (Figure 15). Then, the obtained compound was encapsulated with DSPE-PEG2000 to prepare nanoparticles which were responsive to GSH to release SN38. The in vitro cytotoxicity against human pancreatic carcinoma Panc-1 and BxPC-3 cells of the nanoparticles was remarkably higher than that of irinotecan. Furthermore, the nanoparticles showed a higher inhibitory effect on tumor growth and higher safety than irinotecan in a Panc-1 xenograft model [80].

## 5. Modification of SN38 at C-9 Position and the Opening of E-Ring

The structural modification of SN38 at C-9 position is seldomly reported. Inspired by the structure of irinotecan, Naumczuk inserted azetidine, pyrrolidine and piperidine into SN38 at C-9 position, respectively, to prepare compounds **115**–**119**. These compounds not only inhibited topo 1 but also made DNA alkylation. These compounds showed potent cytotoxicity toward human leukemia HL-60 cells, MCF-7 cells, MDA-MB-231 cells, Caco-2 cells, HT-29 cells and A549 cells comparable to SN38. Compared with the IC_50_ value of SN38 toward normal CRL-179 cells, the IC_50_ values of these compounds, especially compounds **116**, **118** and **119**, were enhanced by around 200-fold, indicating that structural modification at C-9 position endowed SN38 with selectivity to cancer cells [81].

It is well established that the intact E-ring of SN38 is very important for the maintenance of biological activity. Though camptothecin derivatives with opened E-rings were inactive, their carboxylates could undergo lactonization to form the active molecules in the weak acid microenvironment of cancer cells. Hence, ensuring the delivery of the carboxylates of SN38 to tumor tissue is crucial [6]. For example, compound **120** could be adsorbed by cationic supramolecular organic frameworks and effectively delivered to tumor tissue. The half-life of the conversion of compound **120** was 22 h at pH = 6.5, which was shorter than the time gap for repeated administrations of clinically used irinotecan (14 d) or topotecan (21 d) [82]. These findings indicated that the opening of E-rings was not an absolute obstacle to maintain the biological activity of SN38, and ensuring the delivery of SN38 with the opening of E-rings to tumor is curial.

**Figure 16:**
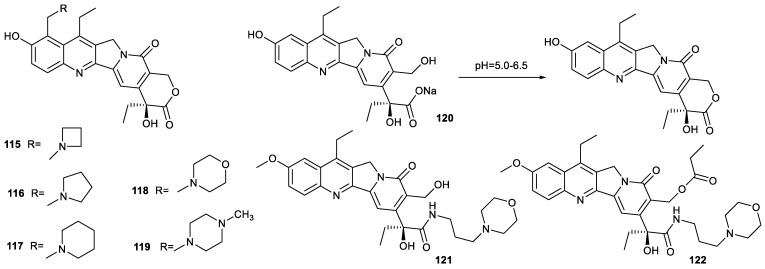
Structures of compounds **115**–**122**.

Compounds **121** and **122** also demonstrated that the opening of the E-ring of SN38 could retain its antitumor activity (Figure 16). Compound **121** was prepared by the amidation of a carboxyl group resulting from the opening of the E-ring of SN38. The compound showed more potent cytotoxicity against A549 cells and HT-29 cells than SN38. Once the hydroxyl group of compound **121** was esterified, the resulting compound **122** only had cytotoxicity comparable to that of irinotecan, indicating once again that the intact E-ring was not indispensable to maintain antitumor activity for SN38 [83].

## 6. Conclusions and Perspectives

Though SN38 has poor water solubility and some side effects, it demonstrates potent antitumor activity and intrinsic fluorescence, which deserves to be further investigated. The aims of the structural modification of SN38 are mainly improving water solubility and enhancing tumor-targeting ability. The conjugation of SN38 with water-soluble molecules and tumor-targeting molecules is an effective strategy to address the above issues. Sometimes, the preparation of more hydrophobic SN38 derivatives for encapsulation by carrier materials is also adopted to improve water solubility. The structural modification of SN38 reported in the literature has been concerned with position 10 and position 20 of SN38 in recent years. Moreover, preparing a prodrug via ester bond is the main form of structural modification of SN38. Modification on other positions such as position 9 and the E-ring of SN38 is rarely reported, owing to a possible decrease in antitumor activity caused by the change in structure. Regardless of how to design the prodrug of SN38, it is crucial for SN38-based prodrugs to be able to quickly release the parent SN38. Hence, the linker between SN38 and other units must be broken to release parent drugs, and the linker responsive to endogenous material in cancer cells is even more noteworthy for designing novel SN38 derivatives, owing to its selectivity for cancer cells.

With the demand for precision medicine, exploiting SN38-based theranostic agents is an important research field due to the intrinsic fluorescence of SN38. However, the wavelength used to excite the fluorescence of SN38 is too short to penetrate deep tissues. Hence, extending the conjugated system of SN38 is worth further investigation on the premise of the maintenance of its antitumor activity. In addition, the resistance and metastasis of cancer cells during chemotherapy is difficult to be cured by a single drug. Hence, designing multitarget drugs for cancer is an important trend for the development of antitumor drugs, which is also applicable for the structural modification of SN38. In summary, the structural modification on SN38 endows its small-molecular derivatives with multifaceted functions, which is beneficial for exploiting novel antitumor drugs.

## Figures and Tables

**Table 1:** Structural Modification of SN38 Reported during 2018–2023.

Types of Structural Modification	Achievable Functions	Current Disadvantages
Modification of C-10 Position	Stimuli-responsive Release of Drugs	Dependent on the type of cell lines or exogenous stimuli
	Tumor Targeting	Dependent on the type of cell lines
	Improvement of Water Solubility	The occuring bottleneck of designing the novel small-molecular SN38 derivatives with improvement of water solubility
	Synergism between Different Drugs	Little consideration of the ratio between drugs
	Theranostics	Weak tissue penetration due to short excitation wavelength; photobleaching
	Other Functions	Null
Modification of C-20 Position	Synergism between Different Drugs	Little consideration of the ratio between drugs
	Tumor Targeting	Dependent on the type of cell lines
	Improvement of Solubility	The occuring bottleneck of designing the novel small-molecular SN38 derivatives with improvement of water solubility
Modification of C-10 Position and C-20 Position	Tumor Targeting	Dependent on the type of cell lines
	Theranostics	Weak tissue penetration due to short excitation wavelength; photobleaching
	Oher Functions	Null
Modification of C-9 Position	Multitarget Inhibition of Cancer Cells	A relatively challenging modification
The Opening of E-ring	Maintenance of Anticancer Activity	The usual reduced anticancer activity

## Data Availability

The data presented in this study are shown in this paper.
